# Peat substrate amended with chitin modulates the N-cycle, siderophore and chitinase responses in the lettuce rhizobiome

**DOI:** 10.1038/s41598-019-46106-x

**Published:** 2019-07-09

**Authors:** C. De Tender, B. Mesuere, F. Van der Jeugt, A. Haegeman, T. Ruttink, B. Vandecasteele, P. Dawyndt, J. Debode, E. E. Kuramae

**Affiliations:** 1 Flanders Research Institute for Agriculture, Fisheries and Food, Plant Sciences Unit, Burgemeester Van Gansberghelaan 92, 9820 Merelbeke, Belgium; 2 Ghent University, Department of Applied Mathematics, Computer Science and Statistics, Krijgslaan 281 S9, 9000 Ghent, Belgium; 3 VIB-UGent Center for Medical Biotechnology, VIB B-9000 Ghent, Belgium; 4 Netherlands Institute of Ecology, department of Microbial Ecology, Droevendaalsesteeg 10, 6708 PB Wageningen, The Netherlands

**Keywords:** Next-generation sequencing, Applied microbiology, Metagenomics

## Abstract

Chitin is a valuable peat substrate amendment by increasing lettuce growth and reducing the survival of the zoonotic pathogen *Salmonella enterica* on lettuce leaves. The production of chitin-catabolic enzymes (chitinases) play a crucial role and are mediated through the microbial community. A higher abundance of plant-growth promoting microorganisms and genera involved in N and chitin metabolism are present in a chitin-enriched substrate. In this study, we hypothesize that chitin addition to peat substrate stimulates the microbial chitinase production. The degradation of chitin leads to nutrient release and the production of small chitin oligomers that are related to plant growth promotion and activation of the plant’s defense response. First a shotgun metagenomics approach was used to decipher the potential rhizosphere microbial functions then the nutritional content of the peat substrate was measured. Our results show that chitin addition increases chitin-catabolic enzymes, bacterial ammonium oxidizing and siderophore genes. Lettuce growth promotion can be explained by a cascade degradation of chitin to N-acetylglucosamine and eventually ammonium. The occurrence of increased ammonium oxidizing bacteria, *Nitrosospira*, and *amoA* genes results in an elevated concentration of plant-available nitrate. In addition, the increase in chitinase and siderophore genes may have stimulated the plant’s systemic resistance.

## Introduction

Chitin is the second most abundant biopolymer in nature. It is a long-chain polymer of N-acetylglucosamine (GlcNAc), a glucose-like molecule. Major sources of chitin are the exoskeleton of arthropods (insects, crustaceans, etc.), the beaks of cephalopods and the eggs and gut linings of nematodes^1^.

In soil, degradation of chitin is mediated by enzymes produced by certain bacteria and several fungi, such as *Streptomyces* and *Trichoderma*^2^. More specifically, the biological degradation of chitin to GlcNAc monomers relies on a catalytic action of hydrolytic enzymes, called chitinases and N-acetylglucosaminidases. In addition, chitin can also be converted to a more soluble biopolymer, chitosan, through deacetylases. The enzymatic action of chitosanases further degrades chitosan into N-glucosamines^3,4^.

Besides its importance as structural polymer, chitin and its deacetylated form chitosan display biological activity. Beneficial effects of both compounds have been reported regarding plant growth and disease resistance. Chitin amendment of a peat substrate promotes growth of lettuce and other plants^5,6^. Soil amended with chitosan promotes plant growth in tomato, soybean sprouts, sweet basil and grapevine^7–10^. The observed increases in plant growth under chitin and chitosan supplementation can be attributed to an increase in plant-available nutrients in the growing medium. Chitin and chitosan have high N content (6.1–8.3%) and are also calcium rich^11,12^. The production of catabolic enzymes for chitin-degradation by micro-organisms in the rhizosphere can release Ca into the growing medium and break down N sources in forms more accessible for uptake by plants and microorganisms. For chitosan, mechanisms besides the provision of plant nutrients may also be present. In studies with orchids, chitosan affects plant growth even at concentrations under the nutritionally effective range^13,14^. The underlying mechanisms of this effect will need to be unraveled. Currently, they are attributed to the stimulation of plant-growth promoting rhizobacteria (PGPR)^15^.

Production of chitin-degradable enzymes not only affects plant growth, but also shows positive effects on plant disease resistance. The production of chitin-catabolic enzymes results in the lysis of chitin-rich cell walls of several pests and pathogens such as insects, pathogenic fungi and plant parasitic nematodes^5,16–18^. In addition, the application of chitin or chitosan in low concentrations in the soil or on the plant leaves can activate the plant’s own defense mechanism, also referred to as induced systemic resistance. In several crops, a number of “chitin elicitor binding proteins” (CEBiP) have been isolated, which enables the plant to respond to chitin oligomers that are either released from pests and pathogens or come from an added source of chitin^19,20^. The oligomers are degraded by chitinase activity^5^. Binding of chitin-oligomers to the CEBiP receptors will eventually activate the plant defense response, where the signal is transported throughout the plant via jasmonates^5,21^. Chitin and chitosan are therefore often reported as PAMPs: “pathogen-associated molecular patterns”^5^.

Debode *et al*. (2016) showed that the addition of chitin in peat substrate increases lettuce growth and reduces the survival of a zoonotic pathogen, *Salmonella enterica*, on lettuce leaves. The reduction in *Salmonella* survival can be related to chitin mediated elicitation of the plant defense response^6^. For both responses, the production of microbial chitin-catabolic enzymes is essential for the release of plant-available nutrients and small chitin-oligomers to the peat substrate. Sequencing of bacterial (16S rRNA gene) and fungal (ITS2 gene region) phylogenetic markers showed that chitin increased the relative abundance of PGPR and other rhizosphere microorganisms reported to be involved in the N-cycle and chitin degradation (e.g., *Cellvibrio*, *Pedobacter*, *Dyadobacter*, *Streptomyces*, *Lecanicillium* and *Mortierella* spp.). This information is based on taxonomy, however; no functional information of the microbiota on rhizosphere in peat substrate with chitin is available so far. Therefore we performed a whole genome shotgun (WGS) metagenomics analysis using the same DNA pool of a previous experiment (Debode *et al*.)^6^ to: (1) confirm taxonomic profiles; (2) analyze the functional microbial profile and focus on the presence of chitinase and chitosanase enzymes; and (3) analyze the microbial functions related with nutrient responses. To evaluate the release of N and other plant-available nutrients to the peat substrate, we also evaluated the nutrient content in unamended and chitin-amended peat substrates.

## Results

### Chemical properties of peat, peat amended with chitin and chitin

After eight weeks of plant growth in chitin-amended peat substrate, there was a significant increase in NO_3_^−^, total mineral N and Ca^2+^ levels and a significant decrease in pH and water-extractable P in the chitin-amended peat substrate.

Pure chitin contained trace elements of water-extractable SO_4_^2−^, Mg, Ca, K and C. Na concentration was similar in the peat, the peat-chitin mixture and the pure chitin, while Cl content was higher in pure chitin than peat-amended with chitin and peat substrate (Table 1; Supplementary Table S1). Chitin contains a high total N content (7.1%/dry matter) but has mineral N concentrations below the detection limit (Tables 1 and S1), indicating that the N was organically bound and thus not readily available to plants.

**Table 1:** Chemical properties and water-extractable nutrient concentrations of peat substrate (PS) and chitin-amended peat substrate (Chitin) samples after 8 weeks of plant growth and pure chitin.

	PS	Chitin	p-value	Pure Chitin
pH-H_2_O*	**6**.**23** ± **0**.**02**	**6**.**03** ± **0**.**07**	**0**.**040**	8.80
EC (µS/cm)	469.50 ± 69.92	634.75 ± 38.05	0.092	157.00
N-NO_3_^−^*** (mg/L)	**64**.**58** ± **21**.**49**	**191**.**13** ± **10**.**80**	**0**.**001**	BDL
N-NH_4_^+^ (mg/L)	9.45 ± 0.60	12.73 ± 1.65	0.287	BDL
Total mineral N** (mg/L)	**69**.**30** ± **24**.**06**	**200**.**68** ± **12**.**53**	**0**.**003**	BDL
SO_4_^2−^ (mg/L)	588.95 ± 73.01	571.30 ± 61.72	0.876	26.00
Cl (mg/L)	20.47 ± 1.05	19.97 ± 3.98	0.921	56.90
P in H_2_O* (mg/L)	**66**.**43** ± **10**.**11**	**33**.**58** ± **8**.**41**	**0**.**046**	BDL
Fe (mg/L)	1.1 ± 0.1	0.60 ± 0.02	0.355	/
Mg (mg/L)	75.55 ± 2.12	101.3 ± 1.11	0.081	1.31
Ca* (mg/L)	**287**.**10** ± **8**.**04**	**393**.**65** ± **3**.**50**	**0**.**050**	3.84
K (mg/L)	79.10 ± 5.40	56.85 ± 2.17	0.788	2.51
Na (mg/L)	35.30 ± 0.99	27.65 ± 0.12	0.202	34.25
C (mg/L)	433.80 ± 10.83	572.95 ± 12.53	0.166	86.11
Si (mg/L)	7.15 ± 0.21	6.95 ± 0.09	0.867	/

Values are averages ± standard errors (n = 4). Significances between treatments are indicated by an asterisk (*<0.05, **<0.01, ***< = 0.001). Elements with levels that could not be detected are indicated as BDL = below detection limit. / = not measured. Concentrations of nutrients for PS and Chitin-amended PS are measured as mg/L substrate. For the pure chitin, this is mg/L extract.

### Taxonomic characterization

Four microbial peat substrate DNA samples per treatment from the study of Debode *et al*. (2016) were selected for WGS metagenomics^6^. The resulting data was initially analyzed using a new platform: Unipept Metagenomics Analysis Pipeline (UMGAP). Compared to other widely used metagenomics pipelines (e.g. MG-RAST, EBI metagenomics)^22–24^, UMGAP uses all sequences for taxonomic analysis instead of merely ribosomal subunits. A total of 112,765,345 reads were obtained and after quality control (QC), 14.3% on average of the sequences were removed. From these quality filtered data, an average of 14.6% of the reads were assigned to a taxon. After normalization, OTUs were clustered in 2,197 genera in the UMGAP count table. As a comparison, the MG-RAST pipeline removed 23.5% of the sequences in the QC step, and RefSeq annotation (SILVA LSU, SSU) embedded in MG-RAST allocated 0.30% of the retained reads to a taxon (Table S2). In total, 1,719 taxa were present in the MG-RAST count table.

**Figure 1:**
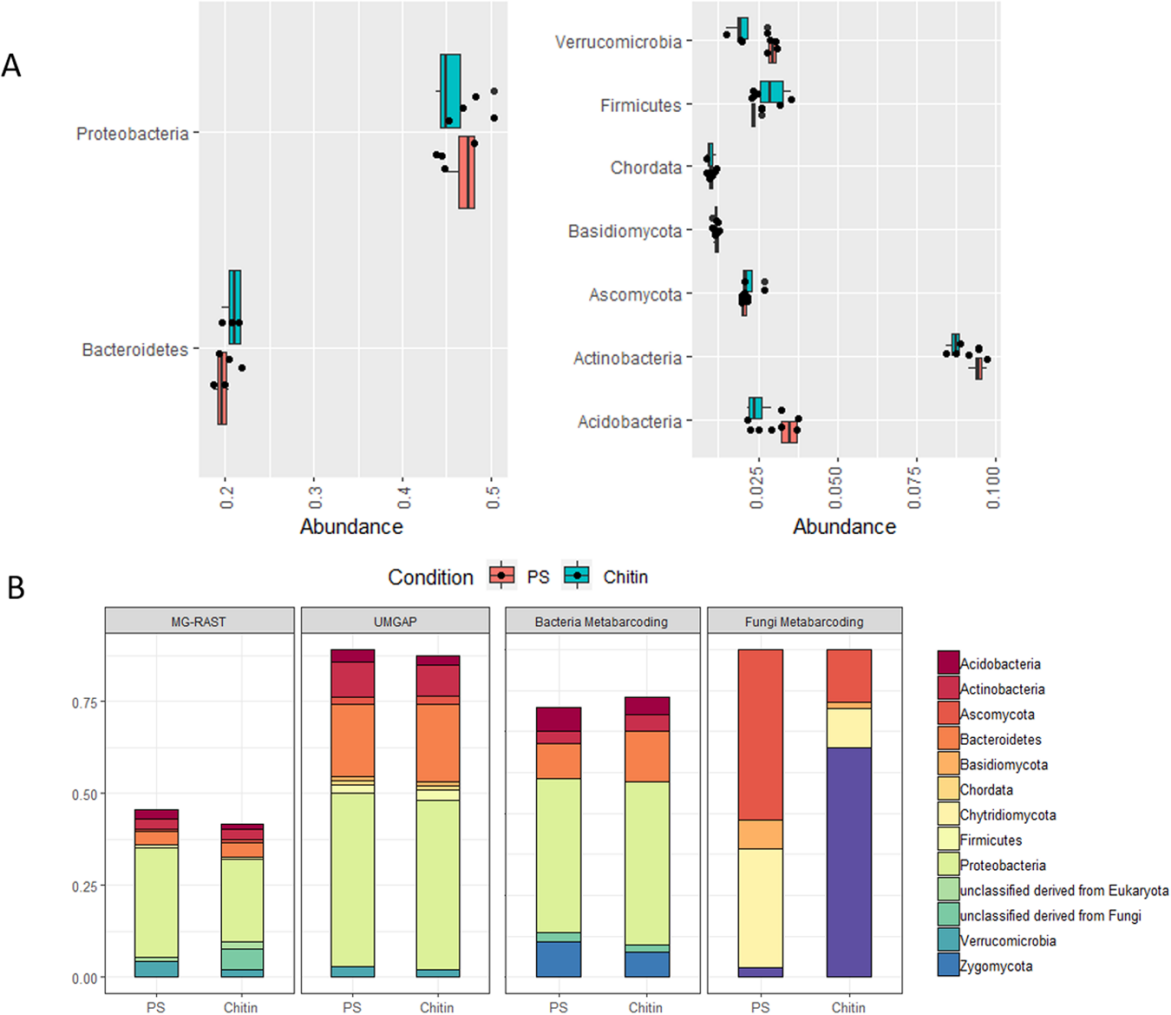
Taxonomical composition of the lettuce rhizosphere microbiome. (**A**) Majorly abundant phyla (relative abundance) in the lettuce rhizosphere which are present for at least 1% in at least one of the treatments (PS = peat substrate, Chitin = peat substrate + 2% chitin). Highly abundant phyla (>10%) are represented at the left, other phyla (1–10%) are represented at the right part of the figure. (**B**) Comparison of the top 10 phyla of the UMGAP data analysis with the conventional MG-RAST analysis. In total, 7 out of the 10 phyla are shared between the two methods. In addition, the abundance of these phyla, with the inclusion of the Zygomycota which contains the *Morteriella* genus in the UNITE database, from the metabarcoding data of Debode *et al*.^6^ is included.

UMGAP analysis showed that independent of chitin addition, Proteobacteria, Bacteroidetes, Acidobacteria, Actinobacteria, Firmicutes and Verrucomicrobia were highly abundant in the samples. (Fig. 1A). Fungi were primarily classified as Ascomycota and Basidiomycota. Other eukaryotic phyla were highly abundant (>2%), i.e., the Chordata. Reads classified as Streptophyta (4.2% ± 2.1%), primarily consisting of OTUs mapped to the lettuce genome, were also found. In comparison, the MG-RAST analysis shares 7 of those phyla in the top 10 ranking (Fig. 1B), and consisted of a rather high amount of reads classified as Streptophyta (52.3% ± 1.9%). As a comparison, data of the previous publication^6^ was added.

Addition of chitin significantly influenced the rhizosphere microbial taxonomic composition (PERMANOVA, p = 0.018). In total, 96 genera were differentially abundant (gene-wise likelihood ratio tests, p-values < 0.05), of which 15 genera had an average abundance of minimum 0.10% in at least one of the two treatments (Table 2). Two fungal genera show the highest increase upon chitin addition: *Hanseniaspora* (18.6x) and *Mortierella* (18.5x), followed by a number of bacterial OTUs, e.g., *Cellvibrio* (7.6x), *Escherichia* (5.0x) and *Nitrosospira* (3.4x).

**Table 2:** Differentially abundant genera (p < 0.05) in the lettuce rhizosphere microbiome cultivated in peat substrate (PS) and peat substrate amended with chitin (Chitin).

Kingdom	Phylum	Genus	PS (%)	Chitin (%)	Increase/Decrease	Potential function*
Archaea	Thaumarchaeota	*Nitrosotalea*	0.30 ± 0.03	0.12 ± 0.01	0.4	Ammonium oxidation^69^
Bacteria	Acidobacteria	*Koribacter*	0.31 ± 0.02	0.17 ± 0.01	0.5	Nitric oxide reduction^70^
	Bacteroidetes	*Pedobacter*	1.02 ± 0.02	1.56 ± 0.15	1.5	PGP and biocontrol^71^
		*Dyadobacter*	0.20 ± 0.01	0.53 ± 0.13	2.6	NA
		*Arachidicoccus*	0.18 ± 0.02	0.39 ± 0.04	2.2	NA
	Proteobacteria	*Nitrosospira*	0.59 ± 0.12	2.04 ± 0.33	3.4	Ammonium oxidation^42,72,73^
		*Cellvibrio*	0.08 ± 0.03	0.58 ± 0.19	7.6	PGP, chitin degradation and N-cycle^25–27^
		*Nitrobacter*	0.19 ± 0.01	0.32 ± 0.02	1.7	Nitrite oxidation^74^
		*Pseudolabrys*	0.24 ± 0.01	0.15 ± 0.01	0.6	NA
		*Escherichia*	0.06 ± 0.01	0.31 ± 0.12	5.0	NA
		*Methylophilus*	0.23 ± 0.07	0.08 ± 0.02	0.4	NA
		*Rhodoplanes*	0.18 ± 0.01	0.10 ± 0.01	0.6	NA
		*Nitrosomonas*	0.06 ± 0.01	0.11 ± 0.02	1.8	Ammonium oxidation^72^
Fungi	Ascomycota	*Hanseniaspora*	0.01 ± 0.01	0.26 ± 0.01	18.6	NA
	Zygomycota	*Mortierella*	0.03 ± 0.01	0.57 ± 0.04	18.5	Chitin degradation^28^ and biocontrol^75^
Protozoa	Ciliophora	*Stylonychia*	0.11 ± 0.02	0.26 ± 0.04	2.5	NA
		*Oxytricha*	0.11 ± 0.02	0.36 ± 0.06	3.4	NA

The average relative abundance of reads (±standard error, n = 4) is shown for plants grown in either in peat or chitin-amended peat. Only genera with an average relative abundance of 0.10% in at least one of the treatments are shown.

*Species of specific genera described in literature involved in the N-cycle, chitin degradation or biocontrol.

NA = To our knowledge, these bacterial genera contain no currently known species involved in the nitrogen cycle, chitin degradation or plant growth promotion.

The abundance of *Salmonella* reads, the zoonotic pathogen added on the leaves, in the rhizosphere of lettuce was not significantly different between peat substrate (0.012 ± 0.001) and chitin-amended peat substrate (0.011 ± 0.001) and was generally low.

### Characterization of functional categories

To study the functional potential of the rhizosphere microbiome, sequences of the shotgun metagenomics data were initially compared to the subsystem database in MG-RAST. In total 39% to 45% of the sequence reads were classified into functional categories (Table S3). At subsystem level 1, the relative abundance of 2 of the 26 functional categories was altered due to chitin addition (Table S4). An increase in category average was observed, namely for iron acquisition and metabolism (p < 0.001), and a decrease was observed in average gene abundance of the nitrogen metabolism (p = 0.002) (Table S4).

At subsystem level 3, a total of 98 functional categories were significantly different: 53 increased and 45 decreased in abundance due to chitin addition (Table S5). These categories could be classified in 22 level 1 categories, with the top 3 being the carbohydrates (n = 11), phages, prophages and plasmids (n = 9) and protein metabolism (n = 9). In most of these level 1 categories, there were both significant increases and decreases of the differentially abundant level 3 functional categories.

One of the carbohydrates categories which increases due to chitin application is named as chitin- and N-acetylglucosamine utilization. This indicates already the presence and induction of chitinase and N-acetylglucosamindase genes in the microbiome of the rhizosphere of lettuce grown in chitin-enriched peat substrate.

Five of the functional categories were clustered within the iron acquisition level. They were linked with either heme uptake, iron uptake, iron scavenging or the production of siderophores. Each of these categories that clustered within iron acquisition showed a significant increase in abundance in the chitin-amended peat substrate. In contrast, from the 98 differentially abundant functional groups, only denitrification could be linked with nitrogen metabolism.

### Chitin-degradation enzyme abundance

**Figure 2:**
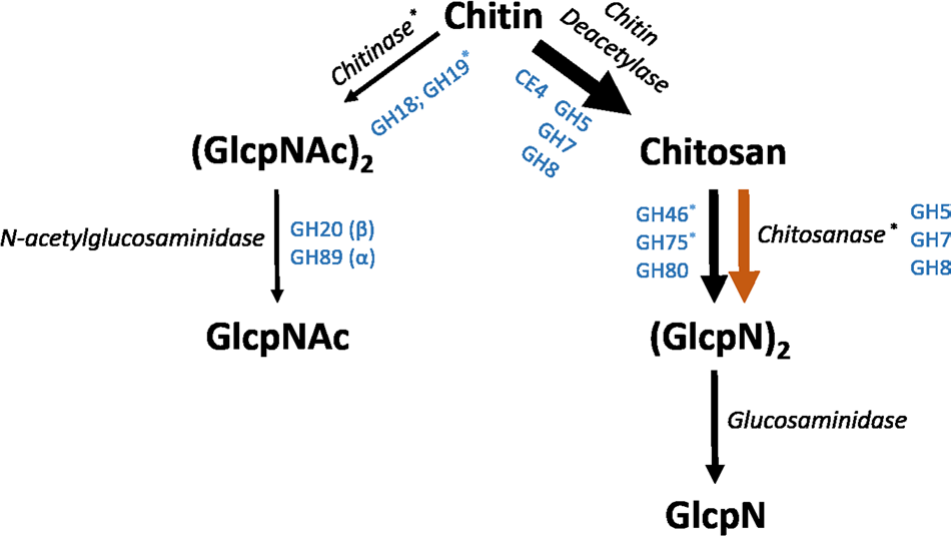
Chitin convergence and degradation pathway. First, chitin can be converted to chitosan through deacetylation and further degraded to N-glucosamines (GlcpN) through glucosaminidase enzymes. Second, chitin can be degraded by chitinases and N-acetylglucosaminidase enzymes to N-acetylglucosamines (GlcpNAc). Enzymes involved in the chitin cycle are classified in glycosyl hydrolase (GH) families and represented in blue. The metagenome reads are mapped towards these enzymes and the RPKG normalized counts representing the number of hits are illustrated by the arrow thickness in the figure. If there was a statistical effect of chitin addition, a second arrow (orange) is drawn representing the normalized count of the genes related to the chitin-added samples.

One of the carbohydrate-related categories that increased in relative abundance due to chitin addition was chitin and N-acetylglucosaminidase utilization (Table S5). To gain deeper insight into chitin metabolism changes, we mapped the metagenome reads to chitosan- or chitin-degradation enzymes. These enzymes are classified in specific glycosyl hydrolase (GH) families. First, the enzymes capable of the deacetylation of chitin to chitosan (CE4, GH5, GH7, GH8), and chitosanases (GH46, GH75, GH80), the enzymes capable of degrading chitosan to N-glucosamines, were mapped (Table S6; Fig. 2). Independent of chitin addition in peat substrate, a high number of metagenome reads were affiliated to chitin deacetylase enzymes and chitosanases (Table S6, Fig. 2). From these chitosanases, only those grouped in GH46 (p < 0.001) and GH75 (p = 0.044) showed a significant increase in abundance due to chitin addition in peat substrate.

Second, the enzymes capable of the degradation of chitin to N-acetylglucosamines (chitinases: GH18, GH19; N-acetylglucosaminidases: GH20) were studied. Independent from chitin addition in peat substrate, the number of reads mapped to chitinases and N-acetylglucosaminidases was 25-times lower than those mapped to chitin-deacetylase and chitosanases. Addition of chitin did increase the potential of chitin degradation to N-acetylglucosamines, however: the number of reads mapped to GH19-related chitinases increased significantly from 0.44 ± 0.05 to 0.84 ± 0.11 (p = 0.0006) (Table S6, Fig. 2). Two genera, *Cellvibrio* and *Mortierella*, that increased in abundance due to chitin addition in peat substrate were previously described to be involved in the chitin-degradation cycle (Table 2)^25–28^.

### Chitin affects the nitrogen cycle

The MG-RAST subsystem classification results showed a reduction in the nitrogen metabolism category in the rhizosphere of lettuce grown in chitin amended peat substrate. To analyze the changes of the nitrogen cycle in further detail, we matched the metagenome reads to profile Hidden Markov Models (HMMs) of 11 nitrification and denitrification genes: *amoA*, *amoB*, *napA*, *nrfA*, *narG*, *nirS*, *nirK*, *norB*, *nosZ*, *nifH* and *ureC* (Table 3). Four other genes involved in the nitrogen cycle (*hao*, *nor*, *nasA*, *nir)* were not studied as they were not available in the FunGene database.

**Table 3:** Potential nitrification and denitrification genes present in rhizosphere of lettuce cultivated in peat substrate (PS) and chitin-amended peat substrates (Chitin).

Enzyme	PS	Chitin	p-value
*amoA AOA*	0.02 ± 0.01	0.01 ± 0.01	0.17
*amoA AOB* ^**^	0.21 ± 0.10	0.66 ± 0.21	0.007
*napA*	1.20 ± 0.05	1.23 ± 0.06	0.53
*narG*	3.13 ± 0.51	2.68 ± 0.17	0.14
*nifH*	0.04 ± 0.02	0.06 ± 0.02	0.18
*nirK*	2.75 ± 0.19	2.82 ± 0.18	0.61
*nirS*	0.05 ± 0.02	0.06 ± 0.04	0.81
*norB*	3.81 ± 0.45	3.43 ± 0.23	0.18
*nosZ* ^**^	1.61 ± 0.18	1.19 ± 0.13	0.0091
*nosZ atypical 1* ^**^	1.76 ± 0.09	1.28 ± 0.14	0.0019
*nosZ atypical 2* ^***^	2.69 ± 0.10	2.01 ± 0.17	0.0005
*nrfA*	0.10 ± 0.01	0.05 ± 0.05	0.08
*ureC*	3.16 ± 0.18	2.96 ± 0.29	0.30

RPKG normalized mean abundance (±standard error) of genes involved in nitrogen cycle.*p-value < 0.05; **p-value < 0.01; ***p-value < 0.001.

**Figure 3:**
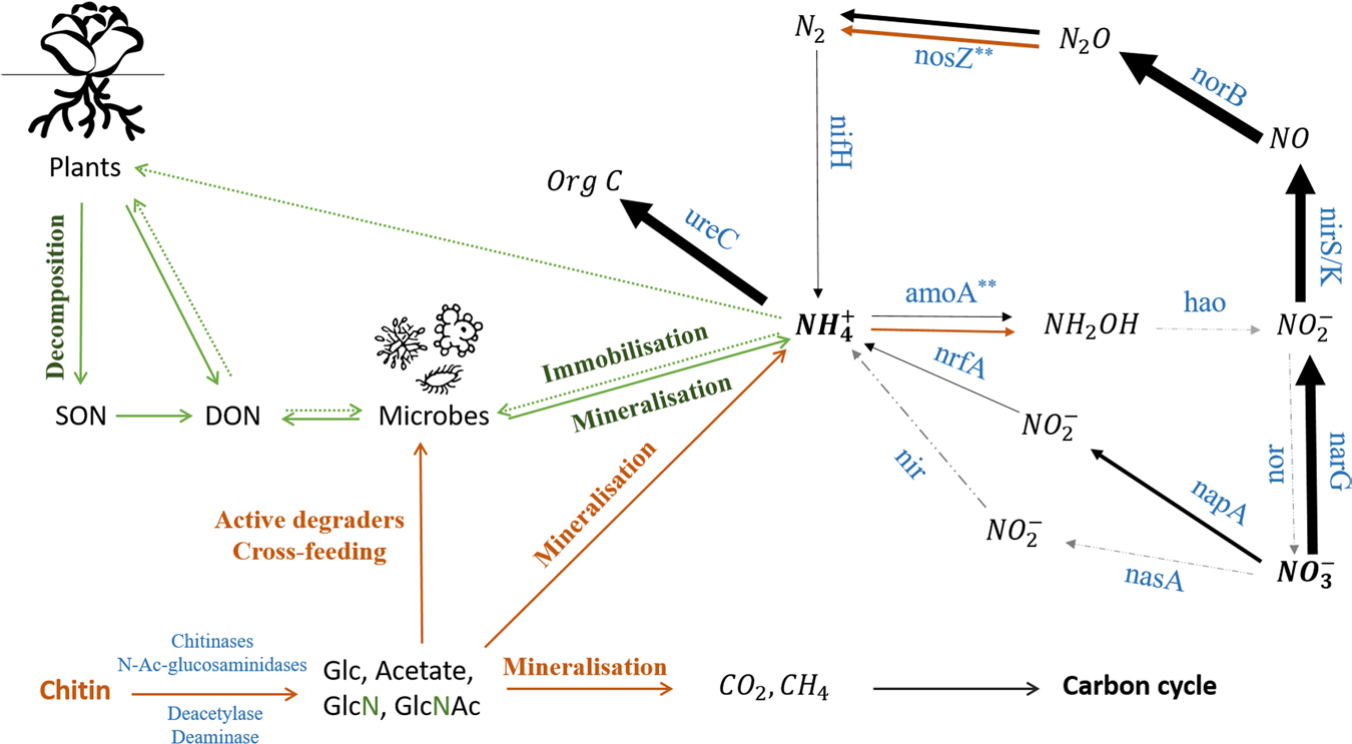
Microbial N-cycle and chitin convergence and degradation pathway mediated through the addition of chitin. Enzymes involved in the N-cycle are indicated in blue. The metagenome reads are mapped towards these enzymes and the RPKG normalized counts representing the number of hits are illustrated by the arrow thickness in the figure. If there was a statistical effect of chitin addition, the enzyme is indicated with an asterisk (*), and a second arrow (orange) is drawn representing the normalized count of the genes related to the chitin-added samples. In addition, the chitin-degradation and its relation with the nitrogen cycle are illustrated in orange. Part of this figure was inspired on the paper of Levy-Booth *et al*.^76,77^.

Based on the normalized counts, a high abundance of genes involved in denitrification was observed compared to the other genes related to nitrogen metabolism. The genes involved for the convergence of nitrate to dinitrogen-oxide (narG, nirS/K, norB) were most abundant in the rhizosphere of lettuce grown in peat as well as in the chitin-amended peat substrate (Fig. 3, Table 3). For the denitrification pathway, chitin only induced a significant reduction of the abundance of nitric oxide signaling genes (*nosZ*) which are involved in converging dinitrogen-oxide to dinitrogen. These genes are classified into three groups, all of which decreased significantly in abundance under chitin supplementation: *nosZ* (p = 0.0091), *nosZ* atypical 1 (p = 0.0019) and *nosZ* atypical 2 (p = 0.0005) (Table 3, Fig. 3).

In addition, there was an increase in bacterial (AOB) ammonium-oxidizing gene (*amoA*, p = 0.007) in the rhizosphere of lettuce grown in chitin-amended peat (Table 3, Fig. 3). In contrast, archaeal (AOA) ammonium oxidizing gene were almost absent in the microbiome grown in peat or in chitin-enriched peat substrate. This increase in ammonium-oxidizing genes was also confirmed by taxonomic data. Within the genera that significantly increased in the rhizosphere after chitin addition, two bacterial ammonium oxidizers (*Nitrosospira*, *Nitrosomonas*) were detected in the UMGAP analysis (Table 2). Especially *Nitrosospira* increased significantly (p = 0.01) from 0.59% ± 0.12 to 2.04% ± 0.33.

## Discussion

Lettuce is worldwide one of the favorite green leafy vegetables^29^. Lettuce can be considered as a high risk food due to the potential presence of human pathogens, such as *Escherichia coli* O157:H7 and *Salmonella enterica* on leaves^30–32^. These zoonotic bacterial pathogens enter the agricultural environment via animal feces, which can contaminate the irrigation water^33^. Human consumption of vegetables contaminated with zoonotic pathogens may lead to disease outbreaks. In a previous study^6^, we showed that chitin addition to the peat substrate promoted lettuce growth and reduced the survival of *S*. *enterica* on lettuce leaves. In this study, reads classified as* Salmonella* were found in low abundance in the lettuce rhizosphere microbiome. These could either be translocated from the lettuce leave to the root, or were already present in the peat substrate. Chitinases and chitosanases enzymes are necessary for chitin degradation. They have been reported to promote plant growth by releasing N and Ca due to chitin breakdown and also to promote the plant’s natural defenses^5,12^. The chitin-amended peat substrate increases the abundance of bacteria and fungi related to chitin degradation in the lettuce rhizosphere^6^. The results of the present study are based on metagenome data, and thus avoid any PCR bias due to amplification of phylogenetic markers. The present results confirm those of Debode *et al*.^6^ as related to the overrepresentation of taxonomic groups related to chitin-degradation such as the fungal genus *Mortierella* (increase of 18-fold) and members of the bacterial genus *Cellvibrio* in the lettuce rhizosphere grown in chitin-amended peat substrate. Differences are observed as well, which can be attributed partly to PCR errors and primer biases within metabarcoding, and differences in taxonomical database (SILVA versus Uniprot) between metabarcoding and metagenomics^34^.

Bacterial genera belonging to the phyla Proteobacteria, Bacteroidetes, Actinobacteria and Firmicutes were highly abundant in the lettuce rhizosphere and have been described to be capable of chitinase production^35,36^. Especially soil-borne bacteria like *Bacillus*, *Pseudomonas* and *Streptomyces* and fungal members are known for their chitin-degrading activity^18^. The fungal member *Mortierella* increased dramatically in abundance relative to the other fungi in the lettuce rhizosphere under chitin-added cultivation^6,28^. This result is reflected in the chitinase genes abundances. Chitinases are either grouped in glycosyl hydrolase (GH) families GH18 or GH19^37^. GH18 chitinase genes are particularly bacterial, while thus far GH19 chitinases have only been found in fungal or *Streptomyces* species^38^. Chitin addition to the lettuce growing substrate only resulted in a significant increase in GH19 family chitinase, indicating that the chitin-effect is especially fungus-related.

The addition of chitin to the peat substrate also resulted in an increase in two glycosyl hydrolase families (GH46, GH75) that contain chitosanases. Independent on the chitin addition to the peat substrate, the abundance of deacetylase enzyme genes was high. These observations indicate that the production of chitosan and further breakdown to N-glucosamines in the lettuce rhizosphere is possible.

The chitin used in this study contains a high amount of fixed total nitrogen (7.1%/dry matter), in agreement with other sources of chitin^39^. Although most of this N is not plant-available a significant higher amount of NO_3_^−^ was measured in the chitin-amended peat substrate after eight weeks of plant growth. Chitin therefore does not provide a direct source of nutrients to plants. It has to be, at least partially, degraded by microbial enzymes, which is decomposed into compounds (e.g., NH_4_^+^, NO_3_^−^ and Ca) available for the plant and finally results in increased plant biomass.

Increased production of chitinases and deaminases leads to an increased degradation of chitin to GlcNAc and GlcN in the peat substrate, which can further be mineralized to CO_2_, CH_4_ and NH_4_^+ ^^38^. A high NH_4_^+^ concentration in soil might be toxic to plants^40^. High amounts of NH_4_^+^ in soils are microbially converted to NO_3_^−^ by nitrification process^41^. NO_3_^−^ is a preferred N source taken up by plants. The nitrification is carried out by a few bacterial and archaeal genera: ammonia oxidation is mediated by the ammonia-oxidizing bacteria (AOB), such as the Betaproteobacteria *Nitrosomonas* and *Nitrosospira* and by the ammonia-oxidizing archaea (AOA), such as the Thaumarchaeota, *Nitrososphaera*^42,43^; nitrite oxidation is carried out by nitrite oxidizing bacteria (NOB), including the Nitrospirae *Nitrospira* and the Alphaproteobacteria *Nitrobacter*^44,45^. Our results show an increase in relative abundance of the genera *Nitrosospira* and *Nitrosomonas* in the rhizosphere of lettuce cultured in chitin-amended peat substrate and an increase in *amoA* AOB genes, which encodes for ammonia monooygenase enzyme and transform ammonium to nitrite. The increase in ammonium oxidizing bacteria and amoA gene, can thus have resulted in the partial conversion of ammonium, produced from chitin-derived GlcNAc and GlcN.

NO_3_^−^ and NO_2_^−^ can also be converted by micro-organisms into N gasses that are no longer available to the plant as nutrient via denitrification. In this study, a decrease in denitrification genes was observed, which could have contributed to the increase in peat substrate nitrate level.

Besides the effect of chitin on the N cycle, there was an increase of the number of genes involved in iron acquisition, and more specifically siderophore production. Microorganisms make use of siderophores to take up iron from the environment^46^. Iron is necessary in many microbial and plant metabolic processes and maintains the cells in healthy state^47^. Iron is required for plant development and Fe deficiency disrupts many plant processes such as respiration or photosynthesis^48,49^. Siderophore production is also linked with an activation of the plant’s induced systemic resistance (ISR). Induced plant resistance by microorganisms, e.g. *Pseudomonas aeruginosa*, is often iron regulated and involves the production of ethylene and jasmonic acid^50,51^.

Based on the above observations, we hypothesize that the lettuce growth promotion by chitin addition is mediated through the following mechanisms. First, the addition of chitin to the peat substrate promotes and activates chitin-catabolic organisms in the lettuce rhizosphere. The production of chitinases and chitosanases leads to the degradation of chitin to plant available N, i.e. NO_3_^−^ and NH_4_^+^. An increase in NH_4_^+^ concentration results in the proliferation and activation of ammonium-oxidizers, which converts ammonium to NO_2_^−^ and NO_3_^−^. Thus, in the presence of chitin-degrading organisms, chitin can act as a nutrient source for plants, particularly N (Fig. 3); however an increase in plant-available Ca was also noted. Ca is important in regulating plant defense responses to pathogens^52^. Second, we observed a high abundance of genes that can deacetylate chitin to chitosan. Chitosan has proven to be an efficient plant growth promotor and can increase fruit yield of several plants, e.g. tomato, okra and oregano by an increase in N or polyphenol uptake^53–55^.

The increase in chitin-degraders and chitinase genes may result in the production of chitin oligomers. These oligomers are able to bind the plant’s CEBIP receptors^5^. In combination with a higher siderophore production, this may have resulted in the induction of the plant’s systemic resistance. Activation of the ISR can also affect pathogens like *Botrytis cinerea* and *Colletotrichum coccodes*, which infect the leaves of the plant^51,56^. The activation of the ISR might therefore have resulted in the reduction of *S*. *enterica* on the lettuce leaves through a response of chitinases and siderophores. The exact mechanism behind this reduction still needs to be unraveled further though.

To conclude, we showed that chitin addition influences the N cycle, especially nitrification by *Nitrosospira* bacteria; increases the number of chitin-degrading organisms; and might play a role in the chitinase and siderophore production. The increase in plant growth is probably due to an increased nutrient availability (NO_3_^−^, NH_4_^+^, and Ca) released as a result of the chitin breakdown by microbial enzymes. The high abundance of siderophore and chitinase genes might indicate an effect on the ISR of the plant.

## Materials and Methods

### Experimental setup

Pelletized butterhead lettuce seedlings (*Lactuca sativa* L. var. capitate “Alexandria”), germinated as described in Debode *et al*.^6^, were planted in a 100% peat based substrate medium pH-H_2_O of 5.5–6.0 (Universal Substrate LP2B, Peltracom, Belgium). Half of the plants were grown in peat substrate, the other half of the plants were grown in 2% chitin-enriched peat substrate. The chitin flakes, purified from crab shells, were obtained from BioLog Hepp Gmbh (lot: 90200705). All pots were placed in a growth chamber at 19 °C/12 °C day/night regime, 70–80% relative humidity and 14 h photoperiod. After 55 days, rhizosphere of six plants per treatment was sampled for total DNA extraction. The DNA was initially used for metabarcoding (Debode *et al*.)^6^ and for shotgun metagenomics (present study) to analyze the potential microbial functions and confirm metabarcoding results on the microbial community composition. Furthermore, samples of the peat substrate were taken for PLFA analysis (see Debode *et al*.)^6^ and chemical characterization, described in the next paragraph. In a previous study^6^, lettuce fresh weight was measured and the survival of *S*. *enterica* or *E*. *coli* on leaves was determined additionally.

### Chemical characterization of growing medium and chitin

In addition to previous research^6^, we determined the chemical composition of the pure chitin flakes and evaluated differences in peat substrate chemical composition due to chitin addition. Total N (Dumas method, ISO 16634-1, Thermo Scientific flash 4000 N analyzer, Massachusetts, United States), organic matter (OM, EN 13039) and P, Ca, K, Mg, Fe, Al and Mn (ashing and digestion with 7 N HNO_3_^−^ (p.a. 65%) and measured with an Agilent 5110 ICP-OES, Santa Clara, USA) concentrations were determined.

Readily available nutrients, electrical conductivity (EC) (EN 13038) and pH in H_2_O (EN 13037) in the pure chitin flakes and in the control and chitin amended peat were measured in a 1:5 soil to water (v/v) suspension. Water-extractable PO_4_-P, Cl, SO_4_ and NO_3_-N were measured with a Dionex DX-3000 IC ion chromatograph (Dionex, Sunnyvale, CA). Water soluble NH_4_-N was measured with a Skalar SAN++ flow analyzer (Skalar Analytical B.V, Breda, The Netherlands). Water-extractable C, Fe, Si, K, Ca, Mg and Na concentrations were measured with ICP-OES.

### Rhizosphere sampling and DNA extraction

The lettuce rhizosphere was sampled according to the protocol of Lundberg *et al*.^57^. Briefly, loose soil was removed from the roots by molding and shaking. Roots were placed in a sterile 50 mL tube containing 25 mL phosphate buffer and shaken in a vortex at maximum speed for 15 s to release most of the rhizosphere soil from the roots. The solution is filtered through a 100 µm nylon mesh cell strainer into a new 50 mL tube to remove both plant parts and large sediment particles. The filtrate was then centrifuged for 15 min at 3,200 × g and 250 mg of the pellet was used for DNA extraction with the PowerSoil DNA isolation kit (Qiagen, Hilden, Germany), according to the manufacturer’s instructions.

### Whole genome shotgun sequencing and sequence processing

In total, four samples were selected from each treatment (no chitin addition, 2% chitin addition) for whole genome shotgun metagenomics. From each sample, 1 µg DNA was sheared by ultrasonication using the covaris M20 at NxtGnt with a set length of 450 bp and a 1 min sonication (Ghent, Belgium). Further preparation of the shotgun libraries was done by Floodlight Genomics LLC (Knoxville, USA). Libraries were sequenced on an Illumina HiSeq 2500 lane (2 × 250 bp) by Macrogen, South-Korea. Demultiplexing of the shotgun data and removal of the barcodes was performed by the sequencing provider.

Data quality control, analyzed by fastqc (http://www.Bioinformatics.babraham.ac.uk/projects/fastqc), showed that TruSeq sequencing adaptors were still present in 10% of the reads. Adaptors were removed by cutadapt^58^. The forward and reverse reads were merged and quality filtered by the program PEAR v.0.9.8^59^. Length cut-off values were set between 100 and 500 bp and a minimum overlap size of 100 bp and quality score threshold of 30 were used for all sequences. The raw sequence data is deposited at ENA (https://www.ebi.ac.uk/ena) under the project “ERP017180”.

### Microbial taxonomy

To determine the taxonomic composition of the microbiomes, we used a new metagenomics pipeline: the Unipept Metagenomics Analysis Pipeline (UMGAP). Unipept^60^ is an open source web application designed for metaproteomics analysis that is now extended for the analysis of whole genome shotgun metagenome samples. UMGAP (version 2017) translated each merged sequence read into the peptide sequences of all six reading frames. Each of the amino-acid 9-mers in these peptides is taxonomically classified using a UniProt-backed index from the Uniprot Knowledgebase (version October 2017). The resulting taxa for each merged sequence read are filtered and aggregated into a single consensus taxon. All analysis of UMGAP were done making use of the reference settings. A comprehensive description of UMGAP’s workflow and a tutorial on the use of Unipept metagenomics is available at https://unipept.ugent.be/clidocs/casestudies/metagenomics.

The outcome of UMGAP OTU count tables was compared to that of MG-RAST v3.6 using RefSeq annotations, including eukaryote, bacteria, archaea and viruses^22^. MG-RAST analysis were done on the merged reads. First, data was preprocessed using SolexaQA to trim low-quality regions. Further on, data are dereplicated making use of a k-mer approach, followed by a removal of artificial duplicate reads by DRISEE (Duplicate Read Inferred Sequencing Error Estimation). Making use of vsearch, ribosomal RNA is identified based on a reduced RNA database (clusterd version of SILVA, Greengenes and RDP) and by a BLAT similarity search, taxonomy is annotated. More information on the pipeline can be found in the MG-RAST tutorial guideline^22^.

### Functional analysis of metagenome data using MG-RAST

The metagenomes were compared to the subsystem database in MG-RAST to retrieve functional information. Count tables on subsystem level 1 and level 3 were created and statistically analyzed with egdeR (see statistical data-analysis).

To study the effect of chitin on the nitrogen cycle we matched the metagenome reads to the HMM profile of 11 nitrification and denitrification genes: *amoA*, *amoB*, *napA*, *nrfA*, *narG*, *nirS*, *nirK*, *norB*, *nosZ*, *nifH*, *ureC*. The HMM profiles of the genes were downloaded from the Functional Gene Repository database (FunGene version 8.3; http://fungene.cme.msu.edu/). The metagenome reads were first translated to protein sequences using Prodigal v2.60^61^. The open reading frames were queried for HMM matches using HMMsearch v3.1b2^62^. From these matches, a count table was constructed containing the matched number per HMM per sample with an e-value of maximum 1e-10 and a coverage of 60%. The HMM matches were normalized through reads per kilobase per genome (RPKG) normalization: number of matched reads / HMM length / Number of genome equivalents^62^. The number of genome equivalents is calculated using MicrobeCensus v1.1.0^63^. By applying RPKG normalization, we accounted for differences in library sizes and gene length biases to enable the comparison between samples and treatments.

In addition, we downloaded the profile HMMs of all carbohydrate enzymes listed on dbCAN^64^. From this set of HMM profiles, we selected glycoside hydrolase families involved in the chitin cycle: GH18, GH19 (chitinases), GH20 (N-acetylglucosaminidases), CE4, GH5, GH7, GH8 (chitin deacetylases), GH46, GH75 and GH80 (chitosanases). The translated metagenome reads obtained by prodigal are queried for HMM matches using HMMsearch. A count table was constructed from the number of matched reads having an e-value threshold of 1e-10 and at least 60% coverage. This count table was normalized by RPKG normalization.

### Statistical data analysis

The differences in chemical composition of peat and chitin-amended peat substrate were checked using general linear models. First, the data was log-transformed, with the exception of pH as this is already a log-transformed variable. Second, we tested the effect of treatment through a general linear model with treatment as the main factor. Linearity, homogeneity of variances and normality were checked by plotting residuals vs fitted values, a QQ-plot of the standardized residuals and a scale-location plot.

To study the effect of treatment (peat substrate, chitin-amended peat substrate) on the taxonomic composition, we made use of the OTU tables derived from UMGAP. First, we applied a PERMANOVA analysis, implemented in the vegan package^65^. PERMANOVA was only applied after looking to homogeneity of variances between groups, using the betadisper function in R^66^. In addition, we studied shifts in the taxonomic genera. To filter out sequencing errors and technical artefacts, only taxonomic categories with a count of four per million in at least four samples were kept for analysis in the OTU table. This abundance (four per million) is based on previous research, while the four samples are chosen based on the number of replicates. In total, 30% of OTUs were removed, consisting primarily of singletons. Normalization of the filtered count table was based on the trimmed mean of m-values (TMM) in which we corrected for effective library size^67^. The counts were then modeled per taxonomic category using a negative binomial (NB) model with the main effect of treatment. The effective library size was used as an offset in the model for normalization purposes; hence the model parameters have an interpretation in terms of changes in relative abundance. Empirical Bayes estimation of the overdispersion parameters of the NB model were adopted using the quantile-adjusted conditional maximum likelihood method. Statistical tests were adopted on the appropriate contrasts of the model parameters. We adopted the Benjamini-Hochberg false discovery rate procedure to correct for multiple testing. After correction, only those genera with a p-value below the significance threshold of 0.05 were seen as statistical significant. All of these analyses were done using edgeR package, version 3.12.0 in R v3.4.2^66,68^.

The effect of treatment (peat substrate, chitin-amended peat substrate) on the potential microbial functions was analyzed on the subsystem level 1 and level 3 tables retrieved from MG-RAST. Only functional categories with a count of four per million in at least four samples were kept for the analysis. Normalization, count modelling, empirical Bayes estimation and statistical tests were performed as described for the taxonomical classification.

To study differences in HMM matched number of reads after normalization for both nitrification and denitrification; and chitin-related genes, counts are modeled using a quasi-Poisson model with the main effect of treatment to correct for overdispersion. Generalized linear models (based on the quasi-poisson model) were applied on the appropriate contrasts of the model parameters, and the Benjamini-Hochberg false discovery rate procedure is used to correct for multiple testing. Genes were considered statistical significant if the p-value was below the significance thresholf of 0.05.

## Supplementary information

Supplementary information

## Data Availability

The raw sequence data of the shotgun metagenomics sequencing is deposited and freely available at ENA (https://www.ebi.ac.uk/ena) under the project “ERP017180”.
