# Supplementary material for: Peat substrate amended with chitin modulates the N-cycle, siderophore and chitinase responses in the lettuce rhizobiome
Source: Sci Rep. 2019 Jul 9;9:9890. doi: 10.1038/s41598-019-46106-x (PMC6617458; doi:10.1038/s41598-019-46106-x)
Supplement: Supplementary file 1 — Supplementary information [file 41598_2019_46106_MOESM1_ESM.pdf]

Supplementary material:

Peat substrate amended with chitin modulates the N-cycle, siderophore and chitinase in the lettuce rhizosphere microbiome

De Tender C[1,2,*], Mesuere B[2,3], Vanderjeugt F[2], Haegeman A[1], Ruttink T[1], Vandecasteele B[1], Dawyndt P[2], Debode J[1], Kuramae E E[4]

Table S1: Total concentrations of nutrients in pure chitin before addition to the growing medium.

**Cd (mg/kg DM)**	0.1
**Cr (mg/kg DM)**	6.1
**Cu (mg/kg DM)**	1.0
**Pb (mg/kg DM)**	0.3
**Ni (mg/kg DM)**	2.9
**Zn (mg/kg DM)**	1.8
**Mn (mg/kg DM)**	2.0
**P (mg/kg DM)**	528.0
**K (mg/kg DM)**	56.7
**Mg (mg/kg DM)**	199.8
**Ca (mg/kg DM)**	2019.0
**Na (mg/kg DM)**	1077.2
**Fe (mg/kg DM)**	143.0
**Al (mg/kg DM)**	34.5
**N (%/ADS)**	7.1

Table S2. Number of sequencing reads per sample. The original number of reads (raw data), the number of reads retained after quality control (QC) and the number of reads aligned to a taxonomical identity (Taxonomy) by MG-RAST and Unipept pipelines. PS = peat substrate; Chitin = peat amended with chitin

Sample	Original number of reads	MG-RAST		UMGAP	
		QC	Taxonomy	QC	Taxonomy
PS2	10,500,768	8,690,797	23,138	9,104,062	1,183,348
PS3	19,721,339	15,703,981	38,888	16,946,110	2,562,961
PS4	10,454,657	8,524,676	22,423	9,060,597	1,341,623
PS5	23,458,499	8,400,028	19,536	20,474,220	2,931,258
Chitin1	10,221,155	8,312,158	20,885	8,884,083	1,339,812
Chitin3	10,331,359	8,461,027	25,499	8,879,723	1,253,102
Chitin5	11,531,506	9,121,736	34,810	9,448,011	1,566,486
Chitin6	16,546,062	13,609,531	56,491	13,900,194	1,962,736

Table S3. Number and percentage of the reads per sample classified as function by MG-RAST. PS = peat substrate; Chitin = peat amended with chitin.

Sample	QC-filtered reads	Function-related sequences	%
**PS2**	8,690,797	3,566,657	41.04
**PS3**	15,703,981	7,068,650	45.01
**PS4**	8,524,676	3,243,322	41.83
**PS5**	8,400,028	3,658,359	43.55
**Chitin1**	8,312,158	3,715,013	44.69
**Chitin3**	8,461,027	3,448,172	40.75
**Chitin5**	9,121,736	3,559,690	39.02
**Chitin6**	13,609,531	5,362,006	39.40

Table S4. Trimmed mean of M-values (TMM) normalized average counts (±standard error, n=4) of functional categories between peat substrate (PS) and chitin amended peat substrate (Chitin) samples. Functional categories of the SEED database level 1 are shown.

**Function**	**PS**	**Chitin**	***P-value***
Amino acids and derivatives	9.73±0.02	9.73±0.09	0.959
Carbohydrates	13.20±0.13	13.02±0.04	0.140
Cell division and cell cycle	0.94±0.01	0.95±0.02	0.688
Cell wall and capsule	4.11±0.03	4.09±0.01	0.428
Clustering-based subsystems	12.49±0.04	12.46±0.07	0.999
Cofactors, vitamins, prosthetic groups, pigments	5.33±0.03	5.31±0.04	0.564
DNA metabolism	4.56±0.02	4.59±0.02	0.469
Dormancy and sporulation	0.11±0.01	0.11±0.01	0.494
Fatty acids, lipids and isoprenoids	2.75±0.05	2.76±0.03	0.707
**Iron acquisition and metabolism**	**0.93±0.02**	**1.03±0.04**	**<0.001**
Membrane transport	4.06±0.05	4.08±0.03	0.633
Metabolism of aromatic compounds	1.79±0.09	1.83±0.06	0.516
Miscellaneous	6.31±0.03	6.35±0.03	0.420
Motility and chemotaxis	1.37±0.01	1.35±0.02	0.268
**Nitrogen metabolism**	**1.37±0.02**	**1.29±0.03**	**0.002**
Nucleosides and nucleotides	3.09±0.04	3.08±0.01	0.444
Phages, prophages, transposable elements, plasmids	1.16±0.03	1.23±0.06	0.057
Phosphorus metabolism	1.27±0.03	1.29±0.02	0.391
Photosynthesis	0.07±<0.01	0.08±0.01	0.054
Potassium metabolism	0.88±0.02	0.88±0.02	0.918
Protein metabolism	7.83±0.01	7.80±0.02	0.622
Regulation and signaling	1.09±0.02	1.11±0.01	0.053
Respiration	4.35±0.02	4.26±0.04	0.057
RNA metabolism	3.68±0.01	3.73±0.04	0.151
Secondary metabolism	0.26±<0.01	0.25±0.01	0.311
Stress response	2.71±0.01	2.75±001	0.242
Sulfur metabolism	1.05±0.01	1.06±0.01	0.657
Virulence, disease and defense	3.43±0.07	3.45±0.11	0.764

Bold = Significant differences.

Table S5. TMM normalized counts of functional categories significantly different in the rhizosphere due to chitin addition in peat substrate.

Level 1	Level 3	PS	Chitin
Amino Acids and Derivatives	Branched chain amino acid degradation regulons	3523±112	3789±17
	Branched chain amino acid biosynthesis	6497±66	6147±87
	Valine degradation	1528±8	1632±30
	Histidine degradation	895±21	108±61
	Methionine degradation	1524±17	1400±34
	Threonine and homoserine biosynthesis	2523±12	2393±18
	Proline, 4-hydroxyproline uptake and utilization	1580±27	1731±56
Carbohydrates	(GlcNAc)2 catabolic operon	146±12	205±16
	Chitin and N-acetylglucosamine utilization	1770±64	2147±65
	Pentose phosphate pathway	3865±63	3554±74
	Pyruvate:ferredoxin oxidoreductase	230±16	148±22
	Lactose and galactose uptake and utilization	1685±39	1524±71
	Lactose utilization	798±25	692±41
	Fermentations: lactate	1813±30	1386±52
	L-ascorbate utilization	45±1	30±4
	Lacto-N-biose I and Galacto-N biose metabolic pathway	593±5	524±23
	Propionyl-CoA to succinyl CoA module	2225±16	1204±28
	Cellulosome	379±19	436±15
	Ethanolamine utilization	630±33	481±14
	Glycerol and glycerol-3-phosphate uptake and utilization	1961±33	2094±45
	Mannitol utilization	372±7	445±26
Cell wall and capsule	Lipid A-Ara4N pathway	2106±92	1886±66
	LOS core oligosaccharide biosynthesis	1337±16	1199±32
	tRNA-dependent amino acid transfers	7±2	13±1
Clustering-based subsystem	CBSS-258594.1.peg.3339	3150±29	2981±54
	CBSS-314269.3.peg.1840	1107±20	931±48
	CBSS-393131.3.peg.612	2417±87	2615±101
	Glycogen metabolism cluster	1955±80	1774±51
	CBSS-316057.3.peg.1308	219±14	277±23
Cofactors, vitamins, prosthetic groups, pigments	Molybdenum_cofactor_biosynthesis	3795±42	3539±56
	Heme_biosynthesis_orphans	100±10	59±4
DNA metabolism	DNA_phosphorothioation	66±6	48±6
	Restriction-Modification_System	1771±30	1623±82
	Type_I_Restriction-Modification	1807±26	1628±108
Dormancy and sporulation	Spore_pigment_biosynthetic_cluster_in_Actinomycetes	10±3	25±5
Fatty acids, lipids and isoprenoids	Fatty_Acid_Biosynthesis_FASI	28±3	45±10
Iron acquisition and metabolism	Heme,_hemin_uptake_and_utilization_systems_in_GramNegatives	764±18	876±30
	Iron_acquisition_in_Streptococcus	115±12	146±14
	Iron_Scavenging_cluster_in_Thermus	108±33	173±43
	Bacillibactin_Siderophore	10±1	18±2
	Siderophore_assembly_kit	221±16	324±36
Membrane transport	ATP-dependent efflux pump transporter Ybh	411±37	273±24
	Periplasmic-Binding-Protein-Dependent Transport System for α-Glucosides	251±14	311±28
	Citrate Utilization System (CitAB,CitH,and tctABC)	7±1	14±1
	Vir-like type 4 secretion system	239±30	316±24
	Predicted_secretion_system_X	130±19	172±21

Metabolism of aromatic compounds	Anaerobic_toluene_and_ethylbenzene_degradation	46±7	31±5
Miscellaneous	Homogentisate pathway of aromatic compound degradation	1181±75	1347±97
	Phenylacetyl-CoA catabolic pathway (core)	1653±2	1901±12
	YaaA	54±9	80±8
	At5g38900	278±8	330±8
	COG0398	493±17	441±11
	COG2078	59±10	32±7
	COG3146_experimental	1362±36	1475±47
	COG3380_COG2907	612±31	697±45
Nitrogen metabolism	Denitrification	1674±120	1378±79
Nucleosides and nucleotides	Ribonucleotide_reduction	3258±70	3336±51
	De_Novo_Pyrimidine_Synthesis	3421±97	3208±45
Phages, prophages, transposable elements, plasmids	Phage_Dual_Exonuclease_Exclusion	3±1	9±2
	Phage_entry_and_exit	101±21	150±20
	Phage_introns	5±1	12±3
	Phage_nin_genes_-_N-independent_survival	2±2	16±11
	Phage_packaging_machinery	228±26	330±76
	Phage_replication	668±30	767±62
	Prophage lysogenic conversion modules	20±7	34±6
	r1t-like_streptococcal_phages	1236±99	1628±260
	Plasmid-encoded_T-DNA_transfer	310±21	373±32
Protein metabolism	Ribosome_LSU_eukaryotic_and_archaeal	35±7	60±12
	Translation_elongation_factor_G_family	1802±31	1689±9
	Proteasome_eukaryotic	33±11	70±14
	Proteolysis_in_bacteria,_ATP-dependent	4197±110	3956±45
	GroEL_GroES	1660±86	1510±34
	Thermosome,_archaeal	14±4	5±1
	Inteins	195±10	160±9
	Selenocysteine_metabolism	444±15	382±21
	Selenoprotein_O	321±5	369±9
Regulation and cell signaling	Cell envelope-associated LytR-CpsA-Psr transcriptional attenuators	40±3	63±6
	Regulatory Intramembrane Proteolysis Pathways	9±1	16±3
	Biofilm_Adhesin_Biosynthesis	33±13	17±1
	Quorum_sensing_in_Yersinia	10±3	17±2
Respiration	CO_Dehydrogenase	1436±55	1219±89
	H2:CoM-S-S-HTP_oxidoreductase	22±5	10±2
	Hydrogenases	1131±44	859±51
	NiFe_hydrogenase_maturation	321±17	260±17
	Carbon_monoxide_induced_hydrogenase	15±4	5±1
	Formate_hydrogenase	4716±140	4190±122
	Mebrane_bound_hydrogenases	13±2	7±2
RNA metabolism	Group_II_intron-associated_genes	542±179	370±55
	Eukaryotic rRNA modification and related functions	90±11	116±9
	Spliceosome	9±1	24±8
	RNA_polymerase_archaeal	30±6	16±3
	RNA_polymerase_I	4±1	13±3
	RNA_polymerase_II_initiation_factors	7±1	17±3
	RNA_polymerase_III	4±2	20±5
Stress response	Synthesis_of_osmoregulated_periplasmic_glucans	3725±64	3340±53
Sulfur metabolism	Sulfate_reduction-associated_complexes	39±2	15±3
Virulence, disease and defense	Marinocine, a broad-spectrum antibacterial protein	8±2	18±1
	Beta-lactamase	2156±60	2317±48

Grey color means increase and while color means decrease in abundance of functional category.

Table S6. RPKG normalized number of reads mapped to enzymes known for chitin conversion or degradation, classified in glycosyl hydrolase (GH) families.

Enzyme category	Glycosyl hydrolase family	Peat	Peat+Chitin	p-value
Chitinases	GH18	0.99±0.11	1.24±0.25	0.11
	**GH19**	**0.44±0.05**	**0.84±0.11**	**0.0006**
	Total chitinases	1.43±0.29	2.09±0.25	
N-acetyl-glucosaminidases	GH20	1.44±0.16	1.51±0.17	0.60
Chitin deacetylase	CE4	26.1±0.52	25.5±0.56	0.15
Chitin deacetylase/ chitosanase	GH5	1.52±0.24	1.60±0.14	0.61
	GH7	0.00±0.00	0.00±0.00	1
	GH8	0.33±0.03	0.42±0.11	0.18
	Total chitosanase/ Chitin deacetylase	1.86±0.23	2.01±0.22	
chitosanase	**GH46**	**1.00±0.21**	**2.12±0.32**	**<2E-16**
	**GH75**	**0.027±0.02**	**0.047±0.3**	**0.04**
	GH80	0.32±0.14	0.32±0.12	0.08
	Total chitosanase	1.45±0.05	2.43±0.36	

Values are mean ± standard deviations (n=4). Bold are Glycosyl hydrolase family statistically significant different
